# Characteristics linked to mortality risk among individuals with drug use disorders enrolled in drug rehabilitation facilities in Japan

**DOI:** 10.1002/pcn5.70112

**Published:** 2025-05-15

**Authors:** Satomi Mizuno, Takuya Shimane, Satoshi Inoura, Maki Kitamura, Toshihiko Matsumoto

**Affiliations:** ^1^ Department of Drug Dependence Research, National Institute of Mental Health National Center of Neurology and Psychiatry Kodaira Tokyo Japan; ^2^ Department of Nursing Faculty of Nursing, Niigata Seiryo University Niigata Japan

**Keywords:** therapeutic community, 12‐step program, mortality risk, drug use disorder, rehabilitation facilities

## Abstract

**Aim:**

This study evaluated survival rates and examined characteristics associated with mortality among individuals with drug use disorder (DUD) enrolled in drug addiction rehabilitation facilities in Japan.

**Methods:**

This longitudinal cohort study, conducted from October 2016 to October 2021, followed 361 individuals with DUD residing in drug addiction rehabilitation centers nationwide. Participants had a mean age of 41.1 years (standard deviation 9.7) and most were men. About half had not completed high school or had criminal records, including drug offenses. Data were collected through eight follow‐up assessments conducted approximately every 6 months. Kaplan–Meier analysis was used to calculate 5‐year survival rates, and age‐specific mortality was evaluated among participants. A Cox proportional hazards model identified characteristics influencing mortality risk, including demographic factors, medical history, and abstinence maintenance. Hazard ratios and 95% confidence intervals were calculated to assess mortality risk.

**Results:**

The survival rate was 95.4%, with 14 deaths recorded. Mortality was highest among participants aged over 60 years. Age and the presence of bloodborne or sexually transmitted infections were significantly associated with higher mortality, while abstinence was associated with reduced mortality.

**Conclusion:**

This study is the first to examine survival rates and mortality factors among individuals with DUD in drug addiction recovery centers in Japan. Findings suggest that these facilities may help prevent drug relapses and reduce mortality risk, while highlighting aging and infections as key factors linked to mortality.

## INTRODUCTION

Drug use disorder (DUD) is a chronic condition characterized by impaired control over drug use, causing significant health, social, and legal consequences.^1^, ^2^, ^3^, ^4^, ^5^, ^6^, ^7^ DUD is also associated with higher risks of physical and mental health problems, social isolation, and premature death.^1^, ^2^, ^6^, ^7^, ^8^ Key risk factors include drug use type and frequency, dependence severity, and psychiatric comorbidities.^9^, ^10^, ^11^ Patients with DUD often die from overdose,^5^, ^12^, ^13^, ^14^ accidents,^15^, ^16^ suicide,^5^ drug use‐related lethal activities, ^17^, ^18^, ^19^, ^20^, ^21^ or cardiovascular conditions, liver failure, and infections.^22^, ^23^, ^24^, ^25^, ^26^ Globally, approximately 64 million out of 292 million drug users experience DUD,^27^ with an estimated 130,000 annual deaths attributed to drug use.^28^

Individuals with DUD have a mortality rate three to 17 times higher than the general population, varying by country, region, and drug type.^13^, ^29^, ^30^, ^31^, ^32^, ^33^, ^34^, ^35^, ^36^ Higher mortality rates have been reported in the United States, the United Kingdom, Spain, Australia, and Denmark, among others, among heroin, cocaine, and illicit drug and opioid users.^13^, ^26^, ^29^, ^31^, ^35^

This elevated mortality risk highlights the need for medical and legal support. Early legal intervention may help prevent DUD and reduce deaths,^37^, ^38^, ^39^, ^40^, ^41^, ^42^, ^43^ but excessively punitive measures may delay treatment, leading to repeated offenses and higher mortality.^17^, ^18^, ^19^, ^20^, ^21^, ^44^, ^45^ Consequently, there is growing international support for medical‐focused policies.^21^, ^45^, ^46^, ^47^ Relatedly, Japan's 2016 introduction of the Partial Suspension of Execution of Sentences system^48^, ^49^ for drug offenders reflected a shift from punitive measures to prioritizing treatment and reintegration.

In Japan, drug addiction recovery facilities, which use internationally recognized methods such as the therapeutic community model and the 12‐step program,^50^, ^51^, ^52^, ^53^ are vital in supporting individuals with DUD, including drug offenders. These programs emphasize developing essential life skills and habits for social reintegration, as well as spiritual recovery. Most facilities are run by former drug users. Participation is voluntary or referral‐based, involving structured programs that emphasize peer support, self‐reflection, and life skill development. Following Japan's Partial Suspension of Execution of Sentences system in 2016,^54^ these facilities now also support individuals on probation. After receiving treatment, most participants leave after approximately 2 years and return to society.

However, survival rates and causes of death among facility users remain unclear. Many users reportedly experience severe DUD, with some under legal supervision and others managing chronic illnesses or mental disorders.^55^, ^56^, ^57^, ^58^, ^59^ Thus, facility users have a high mortality risk. Despite this, most previous research and surveys^55^, ^56^, ^57^, ^58^, ^59^ by the Ministry of Health, Labour and Welfare have focused on health status and social reintegration, neglecting long‐term survival rates and mortality‐related characteristics. The only domestic study investigating survival rates and substance use disorder‐related deaths, conducted 40 years ago, focused on alcohol use disorder,^60^ necessitating updated research to reflect the significant changes in the social, legal, and medical landscape over the past four decades. This could help reduce mortality risks and enable more effective treatment and support for individuals with DID.

This study investigated survival rates and mortality risk factors among drug addiction rehabilitation center (DARC) users in Japan, hypothesizing that severe substance use, comorbidities, legal issues, and unstable living conditions contribute to high mortality risks among these individuals.^13^, ^29^, ^30^, ^31^, ^32^, ^33^, ^34^, ^35^, ^36^ Simultaneously, we considered that medical and social support from facilities could reduce these risks, even in severe DUD cases, helping mitigate their disadvantages. To this end, we conducted a 5‐year follow‐up among current users to assess how demographics, medical history, and abstinence affect survival.

## METHODS

### Data source, study design, and ethical considerations

This study utilized secondary data from a follow‐up survey of DARC users^58^, ^61^ spanning 5 years (October 2016 to October 2021), with assessments at 6‐ to 8‐month intervals. Staff conducted follow‐up surveys through telephone or face‐to‐face interviews approximately every 6 months after the baseline.

The prospective cohort study's design has been detailed elsewhere.^57^, ^58^, ^61^ We visited the rehabilitation centers from July to September 2016 to explain the study's significance and purpose to facility managers and staff. Of 57 nationwide DARC facilities, 46 agreed to participate. After sending documents in advance and revisiting each facility, facility directors informed users about the study and confirmed their willingness to participate. Users provided written informed consent and completed a self‐administered questionnaire, which was collected and mailed to our department.

### Participant selection

**Figure 1:**
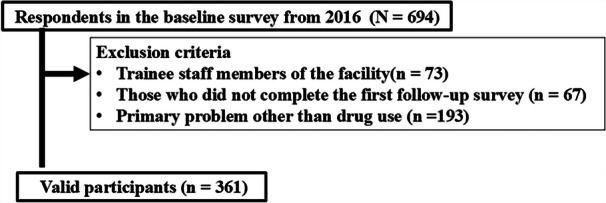
Flowchart of participant selection.

Figure 1 illustrates the flow chart for participant selection. Among the 701 individuals using these 46 facilities, informed consent for participation was obtained from 694 individuals. Of 694 individuals, 361 were included in the analysis based on the following criteria: (1) age 20 years or older, (2) ability to read and write in Japanese, (3) facility user at the baseline survey, and (4) diagnosis of DUD. For consistency, participants were excluded if they were training staff, did not complete the first follow‐up survey, or had nondrug‐related addictions (e.g., alcohol, gambling, or gaming disorders).

### Ethical considerations

The present study received approval from our institution's ethics committee (A2016–022). Our study conforms to the provisions of the Declaration of Helsinki. This study was observational and did not meet the criteria for a clinical trial as defined by the International Committee of Medical Journal Editors. The study involved collecting self‐reported questionnaire data from participants without any interventional component.

### Variables

We collected data for each variable at the start of the follow‐up study using a self‐administered questionnaire completed by participants. Follow‐up surveys were conducted approximately every 6 months through telephone or face‐to‐face interviews by facility staff. If a participant could not be contacted and their absence was confirmed to be due to death, the outcome was recorded as “death.”

For participants who transitioned from inpatient to outpatient care or moved to another residence such as a hospital or prison, follow‐up interviews were conducted via phone calls to their personal mobile phone or place of residence (e.g., home, hospital, prison, temporary accommodation, or shared housing). Survival status was determined based on whether contact was successfully made. Participants who could be reached were classified as alive. If they could not be reached, but their death was confirmed by a family member, acquaintance, or a resident of their place of stay, they were classified as deceased. If neither the participant nor any of their contacts could be reached, their survival status was recorded as undetermined.

Variables were selected based on prior research, focusing on factors potentially related to mortality.^4^, ^5^, ^6^, ^7^, ^8^, ^9^, ^10^, ^11^, ^12^, ^13^, ^14^, ^15^, ^16^, ^17^, ^22^, ^23^, ^24^, ^29^, ^30^, ^31^, ^32^, ^33^, ^34^, ^35^, ^36^, ^62^, ^63^, ^64^, ^65^ Sociodemographic variables, including age, sex, and education level, were assessed. Additionally, gender identity disorder was included, as it can contribute to psychological stress.^66^, ^67^ Criminal history was also recorded.^19^, ^62^ Facility records were used to determine the duration of facility use before the baseline survey (in months). Participants' drug use history was evaluated, covering illegal drugs (e.g., cannabis, methamphetamine, cocaine, heroin, methylenedioxymethamphetamine, and new psychoactive substances), prescription drugs (e.g., hypnotics, anxiolytics, antidepressants, and antipsychotics), and over‐the‐counter drugs (e.g., cough suppressants, cold medicines, pain relievers, and sleep aids). Additionally, prior treatment or support for drug dependence was noted to identify earlier intervention opportunities. The Drug Abuse Screening Test‐20 (DAST‐20) score was recorded when participants began using the facility. This score categorizes dependence into three levels: nonsevere/low (0–5), intermediate (6–10), and severe (11+).^68^ Other key variables included mental health disorders (e.g., mood disorders, schizophrenia, developmental disorders, and eating disorders), chronic diseases (e.g., diabetes, cardiovascular diseases, neurological disorders, and cancer), and bloodborne or sexually transmitted infections (e.g., HAV, HBV, HCV, syphilis, chlamydia, gonorrhea, and HIV), as these significantly impact health risks. Participants' ability to maintain abstinence during the follow‐up period was also analyzed, given its critical importance for health outcomes. Additionally, facility location (urban vs. suburban/rural) was considered to account for regional differences in medical accessibility, and facility staffing levels (seven or more staff) were evaluated for their potential influence on the quality of support provided.

### Statistical analysis

The baseline demographic data of the study participants were summarized using descriptive statistics. Means and standard deviations were calculated for continuous variables, while proportions were used for categorical variables.

Kaplan–Meier survival curves were generated to visually illustrate survival rates over time, along with 95% confidence intervals (CIs). Participants who were lost to follow‐up or stopped using facility services during the observation period were treated as censored, with their observation time included up to the point of loss. Since this study focused on individuals utilizing recovery facilities, participants who discontinued facility use and left were treated as censored data. The mortality rate for each age group (20–29, 30–39, 40–49, 50–59, and 60+ years) was also recorded for the 5‐year period.

Cox proportional hazards regression (HR) with Firth's penalized likelihood method was used to examine the relationship between variables and mortality risk. Initially, all potentially relevant variables identified from prior research were included in the model. A stepwise reduction process was then applied to simplify the model by removing variables with P‐values more significant than 0.05, low statistical significance, or minimal contribution based on *χ* ^2^ statistics. Variables were excluded if their removal did not reduce model performance, as measured by a lower Akaike information criterion (AIC) or an improved likelihood ratio test (LRT) P‐value. Variables identified as essential in previous studies or those with biological relevance (e.g., sex and age) were retained whenever possible, regardless of statistical significance. The final model was refined to align with the study's objectives, minimizing the number of variables to avoid overfitting.

In addition to the primary analysis, a worst‐case scenario was conducted to assess the impact of dropouts. By June 2019 (32 months into follow‐up), four of 46 facilities withdrew, resulting in 96 participant dropouts. The mortality rate was recalculated and survival analysis performed with the assumption that all dropouts had died. The Cox proportional hazards model treated dropouts as deceased cases, and HR was re‐estimated. Kaplan–Meier survival curves were also updated by a comparison of the worst‐case scenario with the original analysis.

Variables statistically associated with mortality risk were included in the final analysis. HRs with 95% CIs were calculated to quantify the impact of each variable on mortality risk. All analyses were conducted using the R software (version 4.2.2).

### Cause‐of‐death classification

Facility staff confirmed participants' survival status during follow‐up surveys. When a participant's death was reported, staff obtained further details from the informant, including the cause of death. We then conducted interviews with facility staff to compile this information. We ascertained the causes of death from individuals close to the deceased.

The reported causes of death were categorized based on the Manual to Fill in a Death Certificate by the Ministry of Health, Labour and Welfare and the International Statistical Classification of Diseases,^69^, ^70^ both commonly used in Japan. The classification included disease‐related deaths, motor vehicle accidents, falls, drowning, smoke‐ and fire‐related injuries, suffocation, poisoning, other accidental external causes, suicide, homicide, unknown external causes, and undetermined causes. These categories were applied to organize the available data on participant deaths systematically. Since determining causes of death was not the primary focus of this study, the information was obtained through verbal reports rather than forensic examinations such as autopsies, therefore the accuracy of the reported causes may be questionable. However, we considered this information valuable for understanding mortality risk factors.

## RESULTS

### Participant characteristics

The average participant age was 41.1 years (standard deviation [SD] 9.7), and most were men (Table 1). More than half the participants had not completed high school, and around half had criminal records, including those related to drug offenses. The average duration of facility use was 31.7 months, with five participants beginning their stay at a DARC during the baseline survey. Most participants reported a history of illegal drug use, and nearly all had received treatment or support before entering the facility. The mean DAST‐20 score was 13.1 (SD 4.1), indicating severe drug abuse. Mental health disorders were reported in 40.7% of participants, 21.1% had chronic illnesses, and 41.0% had a history of infections. Approximately one‐third of the facilities were in rural areas and operated with limited staffing.

**Table 1:** Baseline characteristics of facility users.

	Total (*n* = 361)
Age at baseline (mean [SD] years)	41.1 (9.7)
Biological sex (male)	332 (92.0)
Gender identity differs from biological sex	38 (10.5)
High school education or higher	167 (46.3)
Criminal history	189 (52.4)
Duration of facility use before baseline (mean [SD] months)	31.7 (37.5)
Illegal drug use experience	339 (93.9)
Prescription drug misuse experience	199 (55.1)
Over‐the‐counter drug misuse experience	125 (34.6)
Received treatment or support before starting facility use	356 (98.6)
DAST‐20 score (mean [SD])	13.1 (4.1)
Mental illness	147 (40.7)
Chronic illness	76 (21.1)
History of blood and sexually transmitted diseases	148 (41.0)
Drug abstention during follow‐up	135 (37.4)
Facility location (rural area)	104 (28.8)
Number of facility staff (7 or more)	139 (38.5)

*Note*: Data are presented as *n* (%) unless otherwise indicated.

The average stay at the facility was 31.7 months (SD 37.5). The minimum duration was 0 months, the median was 18 months, and the maximum was 220 months. The interquartile range was 33 months, and the coefficient of variation was 1.2.

Abbreviations: DAST‐20, The Drug Abuse Screening Test‐20; SD, standard deviation.

### Survival analysis and age‐specific mortality rates

**Figure 2:**
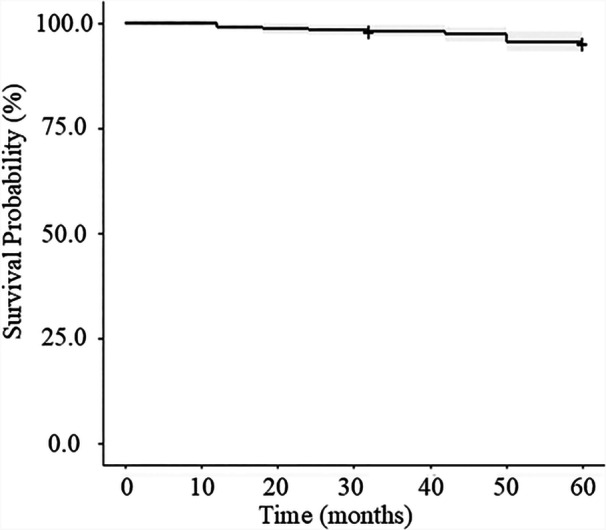
Kaplan–Meier survival curve of facility users showing the estimated survival probability (solid line) over time. The grey‐shaded area represents the 95% confidence interval.

The Kaplan–Meier survival curve (Figure 2 and Table S1) shows a gradual decline, with the survival rate reaching 95.4% (CI 93.0–97.8) at 60 months. The Kaplan–Meier survival curves by age group, showing survival rates at each time point, are presented in Tables S2–6. During the follow‐up period, 14 participants died, with those aged 60 years or older exhibiting the highest mortality rate (20.0%) (Table 2).

**Table 2:** Age‐specific mortality analysis among facility users.

Age group (years)	Total count	Deaths	Mortality rate (%)
20–29	38	1	2.63
30–39	124	1	0.81
40–49	132	5	3.8
50–59	52	4	7.69
60+	15	3	20.0

### Characteristics associated with mortality in addiction recovery facility users

The initial model, which included all variables, had an AIC of 150.7 and an LRT P‐value of 0.001. Variables with low *χ* ^2^ statistics were systematically removed, provided their exclusion did not significantly affect model performance as measured by AIC and LRT. The final model retained three variables: age, infection status, and drug abstinence during follow‐up. This model had an AIC of 158.2 and an LRT *P*‐value of <0.001 (Table 3). Age was significantly associated with a higher mortality risk (HR 1.0689, 95% CI 1.0164–1.1246). Having an infection substantially increased mortality risk (HR 4.0281, 95% CI 1.0820–21.6781), while maintaining abstinence during the follow‐up period provided significant protective effects (HR 0.0379, 95% CI 0.0003–0.2858).

**Table 3:** Cox proportional hazards model results for predictors of mortality.

Model	Variable	HR	95% Cl lower	95% Cl upper	*p* value	*χ* ^2^	AIC	LRT *p* value
Model full	Age at baseline	1.0451	0.9734	1.1338	0.239	1.387	150.7	0.001
	Biological sex	0.5484	0.0039	5.5573	0.676	0.175		
	Gender identity differs from biological sex	2.4680	0.4591	9.8912	―	―		
	High school education or higher	0.3594	0.0635	1.4405	―	―		
	Criminal history	0.3662	0.0766	1.5378	0.175	1.841		
	Duration of facility use before baseline	1.0135	0.8782	1.1810	0.166	1.917		
	Illegal drug use experience	1.0074	0.9967	1.0172	0.537	0.381		
	Prescription drug misuse experience	0.3293	0.0214	47.5971	0.667	0.186		
	Over‐the‐counter drug misuse experience	0.7325	0.1700	3.0562	―	―		
	Received treatment or support before starting facility use	0.6804	0.1112	3.0899	0.550	0.358		
	DAST‐20	0.3390	0.0288	50.5985	―	―		
	Mental illness	1.6947	0.4770	6.3183	0.412	0.673		
	Chronic illness	2.0840	0.5253	8.4333	0.293	1.108		
	Bloodborne or sexually transmitted infections	3.3436	0.8162	18.7225	0.095	2.781		
	Drug abstinence during follow‐up	0.0383	0.0003	0.3176	＜0.001	12.558		
	Facility location	0.6391	0.1484	2.4173	0.518	0.419		
	Number of facility staff	0.5516	0.1248	2.1566	0.398	0.715		
Model 1	Age at baseline	1.0591	0.9924	1.1316	0.0842	2.9811	155.9	0.001
	Biological sex	0.4236	0.0032	3.8580	0.5180	0.4179		
	Criminal history	0.4035	0.0994	1.5357	0.1836	1.7683		
	Duration of facility use before baseline	1.0073	0.9969	1.0170	0.1564	2.0091		
	Chronic illness	1.8963	0.5101	7.0155	―	―		
	Bloodborne or sexually transmitted infections	4.3722	1.1071	24.1757	0.0347	4.4607		
	Drug abstinence during follow‐up	0.0424	0.0003	0.3175	0.0002	13.5118		
Model 2	Age at baseline	1.0646	1.0116	1.1207	0.016	5.749	157.5	<0.001
	Biological sex	0.6723	0.0052	5.4603	0.773	0.83		
	Bloodborne or sexually transmitted infections	4.0188	1.0743	21.6823	0.038	4.298		
	Drug abstinence during follow‐up	0.0379	0.0003	0.2860	<0.001	14.751		
Model final	Age at baseline	1.0689	1.0164	1.1246	0.010	6.695	158.2	<0.001
	Bloodborne or sexually transmitted infections	4.0281	1.0820	21.6781	0.038	4.348		
	Drug abstinence during follow‐up	0.0379	0.0003	0.2858	<0.001	14.783		

*Note*: *p* values and *χ* ^2^ statistics are indicated as “―” for variables where estimation did not converge.

Abbreviations: AIC, Akaike information criterion; CI, confidence interval; DAST‐20, Drug Abuse Screening Test‐20; HR, hazard ratio; LRT, likelihood ratio test.

### Comparison of sensitivity analysis (worst‐case scenario) and standard analysis

In the primary analysis, dropouts were considered alive; in the worst‐case scenario, they were recoded as deceased. The mortality rate increased from 3.9% (14/361) to 30.5% (110/361) under the worst‐case assumption (Table S7). Survival analysis showed a sharp decline at 32 months, coinciding with four facilities withdrawing from the study (Figure S1). In the standard analysis, age and bloodborne/sexually transmitted infections were significant mortality predictors, while drug abstinence was strongly protective (AIC = 158.2). In the worst‐case scenario, age (HR = 1.0176, P = 0.100) and infection status (HR = 0.8282, P = 0.357) lost significance, while drug abstinence (HR = 0.0068, P < 0.001) remained strongly protective (Table S8). The AIC increased to 1279.5, indicating a poorer model fit.

### Cause of death

The causes of death for the 14 deceased participants are summarized in Table S9. The average age at death was 52.6 years and all deceased participants were male. Among them, four deaths (28.6%) were due to cancer. Two participants (14.2%) died by suicide, one of the two suicide cases was due to cancer‐related distress. Two accidental deaths occurred (14.2%), one of which was related to a pre‐existing mental illness. The causes of death for six participants (42.9%) remain unknown due to insufficient information.

## DISCUSSION

This study examined survival rates and factors associated with mortality among 361 users of DARCs in Japan. The cumulative survival rate over the 5‐year follow‐up period was 95.4%, with the highest mortality observed in participants aged 60 years and older. Sustained drug abstinence significantly reduced mortality risk, while age and infections were identified as significant risk factors for mortality.

### Survival rate

The findings of this study revealed a 5‐year survival rate of 95.4% among DARC users, marking the first investigation of mortality rates in this population in Japan. For comparison, using data from the Ministry of Health, Labour and Welfare's *Vital Statistics*,^71^, ^72^ the crude mortality rates for the general population from 2016 to 2020 were converted to estimate a 5‐year cumulative survival rate of approximately 94.7%. Although the survival rate of the general population is an estimate, making direct comparisons challenging, the study suggests that the survival rate of DARC users—who have a high prevalence of severe substance use disorders—may not differ significantly from that of the general population. This contrasts with findings emphasized in earlier studies.^13^, ^29^, ^30^, ^31^, ^32^, ^33^, ^34^, ^35^, ^36^

Given that the study participants were individuals with DUDs utilizing DARC facilities, the results imply that those with DUDs may achieve survival rates comparable to the general population when they have access to appropriate support systems, such as drug addiction recovery programs.^13^, ^29^, ^30^, ^31^, ^32^, ^33^, ^34^, ^35^, ^36^

International studies have reported lower survival rates for individuals with DUDs compared to the general population, with overdose and psychological stress cited as primary contributors to mortality.^13^, ^29^, ^30^, ^31^, ^32^, ^33^, ^34^, ^35^, ^36^ Considering that this study focused on DARC users, a population with a high proportion of individuals experiencing severe DUDs, it was initially hypothesized that survival rates would be low. However, the results showed a 5‐year survival rate, comparable to that of the general Japanese population. This suggests that the support provided by DARC facilities, such as structured rehabilitation programs and community‐based care, may play a critical role in improving survival outcomes.

### Drug abstinence

The finding that sustained abstinence was associated with a reduction in mortality risk suggests that Japan's drug addiction recovery facilities play a critical role in supporting the health of their users. Previous studies have similarly shown that patients with DUDs who receive support exhibit higher survival rates compared to those who do not. Sustained abstinence from drugs has been linked to improvements in both mental and physical health, significantly reducing mortality risks.^37^, ^38^, ^39^, ^40^

Additionally, DARCs operate as therapeutic communities based on the 12‐Step Program, providing a comprehensive support system to maintain abstinence. This system includes residential spaces, emotional support through peer counseling, and referrals to medical institutions.^55^, ^73^, ^74^, ^75^ These features likely contribute to the relatively high survival rates observed among DARC users by creating an environment conducive to abstinence. Improved access to medical care, peer support, and the stability provided by living with others in similar situations could support facility users' health.

Another essential feature of DARCs is the prohibition of alcohol consumption within the facility. Alcohol consumption is known to be associated with drug relapse,^59^ and by banning both drug and alcohol use, DARCs may help reduce cravings for drugs. These findings suggest that Japan's drug addiction recovery facilities may serve as effective support systems in reducing mortality risks among patients with DUD. However, further research should explore the circumstances and characteristics that influence drug withdrawal and its impact on mortality risk.

### Aging

Our results suggest that age impacts mortality risk, with the highest number of deaths observed among facility users aged 60 years and older. These findings align with previous research indicating that the accumulation of health problems associated with aging may increase mortality risk in individuals with DUDs.^76^ While drug overdoses and accidents are the primary causes of death among younger individuals, older users are more likely to die from chronic conditions such as liver and cardiovascular diseases.^77^, ^78^ These changes in health status associated with aging may be a characteristic of those who die while using recovery facilities.

Among older facility users, factors related to aging—such as chronic diseases, reduced physical function, and increased susceptibility to infections—further raise the risk of mortality. For instance, studies have reported that mortality rates increase as health deteriorates among individuals aged 55 years and older, and our results support that trend.^79^ Conversely, the relatively low number of deaths among younger facility users may indicate that the support systems in drug addiction recovery facilities help mitigate early mortality risks. Staff and peers may play a role in preventing emergencies, such as drug overdoses and accidents.

### Bloodborne or sexually transmitted infection

This study demonstrated that bloodborne and sexually transmitted infections were significantly associated with mortality risk among users of drug addiction recovery facilities in Japan. This finding suggests that infections may be a significant risk factor for mortality in facility users. Previous studies have indicated that infections such as HIV and hepatitis are among the leading causes of death among individuals with drug dependence.^22^, ^23^, ^24^, ^80^ Without appropriate treatment, these infections can lead to health deterioration through immune suppression and liver dysfunction, ultimately increasing the risk of death.^81^ The high mortality risk observed in users with a history of infections may have been influenced by delays or interruptions in treatment before facility admission. Many participants had received treatment or support for DUDs; however, they may have faced challenges in continuing regular visits to medical institutions for infection treatment. Previous research has reported that individuals with drug dependence often encounter difficulties in maintaining regular medical care owing to financial hardships, the burden of medical expenses, social stigma, and the apathy or cognitive decline associated with drug use.^82^ Moreover, many facility users were under legal supervision, possibly prioritizing legal actions over infectious disease treatments. Infections can exacerbate mortality risk not only through physical impacts but also by intensifying psychological burdens via social stigma, mental stress, and financial strain. A previous study showed that drug‐dependent individuals infected with HCV or HIV exhibit higher levels of anxiety and depression and a lower quality of life.^83^ These psychological burdens may also have contributed to the mortality observed in facility users.

Our results suggest an association between infection and mortality; however, the wide 95% CI (1.082–21.678) indicates uncertainty regarding the magnitude of this effect. This finding underscores the need for further research to obtain more precise estimates, therefore these values should be discussed in the context of their uncertainty and future studies should explore this effect in greater detail.

This study did not find a significant association between chronic or mental health disorders and mortality risk. Previous research^15^, ^64^ has reported that patients with coexisting chronic or mental health disorders face higher mortality risks compared to those without such conditions. However, in this study, the lack of association may be attributed to appropriate interventions by facility staff, which could have mitigated the impact of these disorders on mortality. Additionally, the timely transfer of participants with worsening chronic or mental health conditions to hospitals or other care facilities might have reduced the apparent relationship with mortality risk. Nevertheless, the lack of detailed data on this study's small number of outcomes presents limitations. These results may reflect constraints due to the sample size or confounding factors such as infections or age. Further research is needed to clarify these findings and understand the factors influencing mortality risks among facility users.

### Causes of death

Most deceased facility users died due to illness or illness‐related suicide or accidents. In many cases, their conditions were already in advanced stages when they sought medical attention, making treatment difficult. This suggests that delayed disease detection and limited access to medical care are critical issues among individuals with DUD. This finding was likely due to differences in living conditions before entering recovery facilities compared to the general population. DUD might be associated with characteristics unique to individuals with DUD, including drug criminals—fewer opportunities for regular health checkups in the workplace, financial difficulties preventing access to medical care, a greater focus on mental health symptoms over physical health concerns, and legal restrictions limiting access to basic healthcare services, for example. Regular health checkups and early medical intervention through independent medical visits are highly effective for disease prevention in the general population. However, for individuals with DUD, these conventional approaches may not be as practical due to their unique characteristics and living conditions. Therefore, we might consider proposing some possible improvements intended for recovery facility users to maintain the effectiveness of recovery programs while ensuring proper management of overall health and mental well‐being, beyond DUD treatment.

### Limitations

This study had several limitations. The sample size may have affected the statistical power; however, this is Japan's first large‐scale national cohort study, surpassing previous research that involved much smaller samples of about 30 participants from one to five facilities.^84^ Second, the 5‐year follow‐up period may only partially capture long‐term outcomes. Nonetheless, it represents the most extended follow‐up of drug addiction rehabilitation facility users in Japan. Third, reliance on self‐reported data introduces potential biases that could affect reliability. Additionally, of the 361 participants, 96 were lost to follow‐up due to the withdrawal of four recovery facilities from the study at 32 months, rather than personal reasons or health deterioration. To address the potential underestimation of mortality, we conducted a worst‐case scenario analysis with the assumption that all 96 dropouts had died. This increased the mortality rate, with a sharp survival decline at 32 months. However, survival trends remained similar before and after this point, suggesting consistent overall patterns. The model fit worsened (AIC 158.2–1279.5), reflecting the extreme nature of the assumption. While this scenario may not fully reflect reality, it allowed us to assess the potential impact of lost‐to‐follow‐up cases and evaluate the robustness of our findings. Finally, a notable limitation is the lack of precise cause‐of‐death data, such as autopsy.

## CONCLUSIONS

The survival rate of those using drug dependence recovery facilities in Japan was 95.4%. This study identified the characteristics of individuals who died while using drug dependence recovery facilities in Japan. Drug abstinence was associated with lower mortality, while older age and a history of infectious diseases were linked to higher mortality. These findings indicate that users of drug dependence recovery facilities have a lower mortality rate and highlight the significant role of these facilities in improving survival outcomes for individuals with drug dependence.

## AUTHOR CONTRIBUTIONS

Takuya Shimane designed the preliminary study. Takuya Shimane, Satoshi Inoura, and Maki Kitamura recruited participants and collected data. Takuya Shimane established the database of research participants. Toshihiko Matsumoto obtained funding. Satomi Mizuno designed the study, performed statistical analyses, and wrote the manuscript. Takuya Shimane, Satoshi Inoura, Maki Kitamura, and Toshihiko Matsumoto supervised the manuscript writing. Satomi Mizuno wrote the initial draft manuscript. All authors revised and contributed to the final version of the manuscript. All authors read and approved the final manuscript submitted for publication.

## CONFLICT OF INTEREST STATEMENT

The authors declare no conflicts of interest.

## ETHICS APPROVAL STATEMENT

The study protocol was reviewed and approved by the Ethics Committee of the National Centre of Neurology and Psychiatry (NCNP) in Japan (A2016–022). Furthermore, written informed consent was obtained from all participants. Our study conforms to the provisions of the Declaration of Helsinki.

## PATIENT CONSENT STATEMENT

Patients received an explanation of the study's purpose and interested patients could participate in the study after providing written informed consent. This study is a secondary analysis of data collected in the original study. Participants had already provided informed consent at the time of the original study, and this analysis uses that existing data.

## CLINICAL TRIAL REGISTRATION

N/A.

## Supporting information

Supporting information 20250321.

## Data Availability

Data are unavailable to protect the confidentiality of study participants.
