# Supplementary material for: Characteristics linked to mortality risk among individuals with drug use disorders enrolled in drug rehabilitation facilities in Japan
Source: PCN Rep. 2025 May 15;4(2):e70112. doi: 10.1002/pcn5.70112 (PMC12079023; doi:10.1002/pcn5.70112)
Supplement: Supplementary file 1 — Supporting information 20250321. [file PCN5-4-e70112-s001.docx]

**Supporting information**

**Supplementary Figure 1**


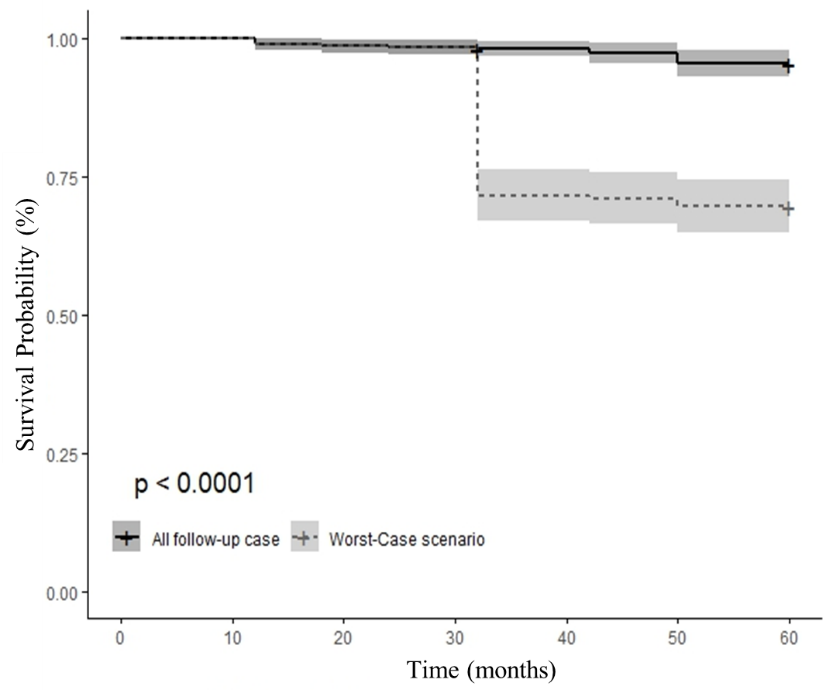
**Comparison of the Kaplan-Meier survival curve between all follow-up cases and the worst-case scenario**

The Kaplan-Meier survival curve shows the estimated survival probability for all follow-up cases (solid line) and in the worst-case scenario (dotted line) over time. The grey-shaded area represents the 95% confidence interval.

**Supplementary Table 1**

**Survival rate at each time point for all participants**

|  |  |  |  |  |  |
| --- | --- | --- | --- | --- | --- |
| Follow-up time (months) | Number at risk | Deaths | Survival rate (%) | 95% Cl lower | 95% Cl upper |
| 6 | 361 | 0 | 100.0 | 100.0 | 100.0 |
| 12 | 361 | 4 | 98.9 | 97.8 | 100.0 |
| 18 | 357 | 1 | 98.6 | 97.4 | 99.8 |
| 24 | 356 | 1 | 98.3 | 97.0 | 99.7 |
| 32 | 355 | 1 | 98.1 | 96.6 | 99.5 |
| 42 | 258 | 2 | 97.3 | 95.6 | 99.1 |
| 50 | 256 | 5 | 95.4 | 93.0 | 97.8 |
| 60 | 251 | 0 | 95.4 | 93.0 | 97.8 |

Abbreviations: CI, confidence interval

**Supplementary Table 2**

**Survival rate at each time point for participants in their 20s**

|  |  |  |  |  |  |
| --- | --- | --- | --- | --- | --- |
| Follow-up time (months) | Number at risk | Deaths | Survival rate (%) | 95% Cl lower | 95% Cl upper |
| 6 | 38 | 0 | 100.0 | 100.0 | 100.0 |
| 12 | 38 | 0 | 100.0 | 100.0 | 100.0 |
| 18 | 38 | 0 | 100.0 | 100.0 | 100.0 |
| 24 | 38 | 1 | 97.4 | 92.4 | 100.0 |
| 32 | 37 | 0 | 97.4 | 92.4 | 100.0 |
| 42 | 24 | 0 | 97.4 | 92.4 | 100.0 |
| 50 | 24 | 0 | 97.4 | 92.4 | 100.0 |
| 60 | 24 | 0 | 97.4 | 92.4 | 100.0 |

Abbreviations: CI, confidence interval

**Supplementary Table 3**

**Survival rate at each time point for participants in their 30s**

|  |  |  |  |  |  |
| --- | --- | --- | --- | --- | --- |
| Follow-up time (months) | Number at risk | Deaths | Survival rate (%) | 95% Cl lower | 95% Cl upper |
| 6 | 124 | 0 | 100.0 | 100.0 | 100.0 |
| 12 | 124 | 0 | 100.0 | 100.0 | 100.0 |
| 18 | 124 | 0 | 100.0 | 100.0 | 100.0 |
| 24 | 124 | 0 | 100.0 | 100.0 | 100.0 |
| 32 | 124 | 0 | 100.0 | 100.0 | 100.0 |
| 42 | 92 | 1 | 98.9 | 96.8 | 100.0 |
| 50 | 91 | 0 | 98.9 | 96.8 | 100.0 |
| 60 | 91 | 0 | 98.9 | 96.8 | 100.0 |

Abbreviations: CI, confidence interval

**Supplementary Table 4**

**Survival rate at each time point for participants in their 40s**

|  |  |  |  |  |  |
| --- | --- | --- | --- | --- | --- |
| Follow-up time (months) | Number at risk | Deaths | Survival rate (%) | 95% Cl lower | 95% Cl upper |
| 6 | 132 | 0 | 100.0 | 100.0 | 100.0 |
| 12 | 132 | 1 | 99.2 | 97.8 | 100.0 |
| 18 | 131 | 0 | 99.2 | 97.8 | 100.0 |
| 24 | 131 | 0 | 99.2 | 97.8 | 100.0 |
| 32 | 131 | 0 | 99.2 | 97.8 | 100.0 |
| 42 | 95 | 1 | 98.2 | 95.7 | 100.0 |
| 50 | 94 | 3 | 95.1 | 90.9 | 99.4 |
| 60 | 91 | 0 | 95.1 | 90.9 | 99.4 |

Abbreviations: CI, confidence interval

**Supplementary Table 5**

**Survival rate at each time point for participants in their 50s**

|  |  |  |  |  |  |  |
| --- | --- | --- | --- | --- | --- | --- |
| Follow-up time (months) | Number at risk | Deaths |  | Survival rate (%) | 95% Cl lower | 95% Cl upper |
| 6 | 52 | 0 |  | 100.0 | 100.0 | 100.0 |
| 12 | 52 | 3 |  | 94.2 | 88.1 | 100.0 |
| 18 | 49 | 0 |  | 94.2 | 88.1 | 100.0 |
| 24 | 49 | 0 |  | 94.2 | 88.1 | 100.0 |
| 32 | 49 | 0 |  | 94.2 | 88.1 | 100.0 |
| 42 | 37 | 0 |  | 94.2 | 88.1 | 100.0 |
| 50 | 37 | 1 |  | 91.2 | 84.1 | 99.9 |
| 60 | 36 | 0 |  | 91.2 | 84.1 | 99.9 |

Abbreviations: CI, confidence interval

**Supplementary Table 6**

**Survival rate at each time point for participants aged 60 and over**

|  |  |  |  |  |  |
| --- | --- | --- | --- | --- | --- |
| Follow-up time (months) | Number at risk | Deaths | Survival rate (%) | 95% Cl lower | 95% Cl upper |
| 6 | 15 | 0 | 100.0 | 100.0 | 100.0 |
| 12 | 15 | 0 | 100.0 | 100.0 | 100.0 |
| 18 | 15 | 1 | 93.3 | 81.5 | 100.0 |
| 24 | 14 | 0 | 93.3 | 81.5 | 100.0 |
| 32 | 14 | 1 | 86.7 | 71.1 | 100.0 |
| 42 | 10 | 0 | 86.7 | 71.1 | 100.0 |
| 50 | 10 | 1 | 78.0 | 58.6 | 100.0 |
| 60 | 9 | 0 | 78.0 | 58.6 | 100.0 |

Abbreviations: CI, confidence interval

**Supplementary Table 7**

**Survival rate at each time point in the worst-case scenario**

|  |  |  |  |  |  |
| --- | --- | --- | --- | --- | --- |
| Follow-up time (months) | Number at risk | Deaths | Survival rate (%) | 95% Cl lower | 95% Cl upper |
| 6 | 361 | 0 | 100.0 | 100.0 | 100.0 |
| 12 | 361 | 4 | 98.9 | 97.8 | 100.0 |
| 18 | 357 | 1 | 98.6 | 97.4 | 99.8 |
| 24 | 356 | 1 | 98.3 | 97.0 | 99.7 |
| 32 | 355 | 97 | 71.5 | 67.0 | 76.3 |
| 42 | 258 | 2 | 71.0 | 66.4 | 75.8 |
| 50 | 256 | 5 | 70.0 | 64.9 | 74.4 |
| 60 | 251 | 0 | 70.0 | 64.9 | 74.4 |

Abbreviations: CI, confidence interval

**Supplementary Table 8**

**Cox proportional hazards model results for mortality predictors in the worst-case scenario**

|  | **Variable** | **HR** | **95% Cl lower** | **95% Cl upper** | **p-**  **value** | **AIC** | **LRT p-value** |
| --- | --- | --- | --- | --- | --- | --- | --- |
| **Worst-case scenario** |  |  |  |  |  | 1279.5 | < 0.001 |
|  | Age at baseline | 1.0176 | 0.9966 | 1.0390 | 0.100 |  |  |
|  | Bloodborne or sexually transmitted Infections | 0.8282 | 0.5494 | 1.2334 | 0.357 |  |  |
|  | Drug abstinence during follow-up | 0.0068 | 0.0005 | 0.2858 | < 0.001 |  |  |

**Supplementary Tabel 9**

**Causes of death among deceased participants (n = 14)**

| ID | Age  at death | Sex | Cause of death | Details of the circumstances leading to death and the place of death |
| --- | --- | --- | --- | --- |
| 1 | 41 | Male | Information not available |  |
| 2 | 68 | Male | Disease | After experiencing health issues while using the facility, the individual visited a hospital and was diagnosed with terminal esophageal cancer. He was subsequently admitted to the hospital and began treatment but he passed away in the hospital three months after admission. |
| 3 | 68 | Male | Suicide | While using the facility, the individual stayed out overnight without permission and died by jumping from an apartment building. The body was found on the street next to the building, and the death was discovered when a passerby reported it to the police. |
| 4 | 47 | Male | Information not available |  |
| 5 | 49 | Male | Information not available |  |
| 6 | 49 | Male | Information not available |  |
| 7 | 58 | Male | Information not available |  |
| 8 | 60 | Male | Disease | The individual had a pre-existing condition of emphysema and experienced respiratory distress while using the facility. He was transported to the hospital by emergency services but passed away two days later. The cause of death was pneumonia. |
| 9 | 61 | Male | Disease | While using the facility, the individual developed a persistent cough and, following the staff's recommendation, visited a hospital. A sarcoma was discovered in the chest, and the cancer was already at a terminal stage. Surgery was not chosen, and the individual remained in the hospital for palliative care until death. |
| 10 | 54 | Male | Disease | The individual had chronic conditions such as diabetes and hypertension, along with drug use disorder and another psychiatric illness, requiring regular medical visits and blood tests. One day, after complaining of severe back pain, he visited a hospital, where liver cancer with bone metastasis was diagnosed. He was immediately hospitalized and started treatment, but since the cancer was already at a terminal stage, a full recovery was not possible, and he passed away in the hospital. According to the medical records, previous blood tests during outpatient visits had shown no abnormalities in liver function, leading to a delayed cancer diagnosis. |
| 11 | 55 | Male | Asphyxiation | The individual had schizophrenia and was classified as having level-2 mental disability. During his time at the facility, his mental condition gradually worsened, leading to increasingly erratic behavior and frequent self-harm and violent incidents. As a result, he was urgently admitted to a psychiatric hospital. While in a protective room at the hospital, he was found by hospital staff in a collapsed state, having choked on their prescribed medication, and was later pronounced dead. The hospital reported the case to the police. The police determined that the cause of death was an accidental choking incident. |
| 12 | 46 | Male | Information not available |  |
| 13 | 30 | Male | Poisoning | While using the facility, the individual frequently stayed out overnight without permission and was on an extended unauthorized absence in the months before his death. During this time, he remained in contact with the facility, wanting to return. However, while away, he overdosed on an antipsychotic drug. Found in a comatose state, he was transported to the hospital but later pronounced dead. The facility learned of his death through police notification. |
| 14 | 50 | Male | Suicide | After transitioning from residential to outpatient care, the individual developed depression. Later, he was diagnosed with an esophageal aortic aneurysm, and a recurrence of pharyngeal cancer was also detected. These diagnoses caused him significant distress. Approximately six months after the illnesses were discovered, he committed suicide by jumping from his residential building. |

Cause of death was classified based on family reports or facility staff input.

Six cases remain undetermined due to lack of verifiable information.
